# Palliative Care in Chronic Heart Failure: A Systematic Review of Its Impact on Symptoms, Quality of Life, and Decision-Making Process

**DOI:** 10.3390/diseases13120389

**Published:** 2025-12-01

**Authors:** Tatiana Dramba, Andrei-Emilian Popa, Mihaela Poroch, Gema Bacoanu, Irina Mihaela Esanu, Elena Popa, Vladimir Poroch

**Affiliations:** 1 Faculty of Medicine, Grigore T. Popa University of Medicine and Pharmacy Iasi, 700115 Iasi, Romania; tatiana.dramba@email.umfiasi.ro; 2 Preventive Medicine and Interdisciplinarity Department, Grigore T. Popa University of Medicine and Pharmacy Iasi, 700115 Iasi, Romania; boanca.mihaela@umfiasi.ro (M.P.); elena.popa@umfiasi.ro (E.P.); 3 2nd Internal Medicine Department, Grigore T. Popa University of Medicine and Pharmacy Iasi, 700115 Iasi, Romania; gema.bacaoanu@umfiasi.ro (G.B.); vladimir.poroch@umfiasi.ro (V.P.); 4 1st Internal Medicine Department, Grigore T. Popa University of Medicine and Pharmacy Iasi, 700115 Iasi, Romania; irina.esanu@umfiasi.ro

**Keywords:** heart failure, palliative care, quality of life, symptom management, advance care planning, systematic review

## Abstract

Background: Palliative care has emerged as a key component in the management of chronic heart failure, addressing persistent physical and psychosocial symptoms that often remain insufficiently controlled by conventional cardiology. This systematic review aimed to evaluate the impact of palliative care interventions on symptom burden, quality of life (QoL) and decision-making processes in adults with chronic heart failure. Methods: A systematic search of PubMed, Scopus and Web of Science identified studies published between January 2005 and February 2025. Eligible designs included randomized controlled trials, observational cohorts and qualitative studies. The review followed PRISMA 2020 guidelines. Methodological quality was assessed using the Cochrane Risk of Bias 2.0 (RoB 2.0), Risk of Bias in Non-Randomized Studies of Interventions (ROBINS-I) and Joanna Briggs Institute (JBI) appraisal tools. Due to heterogeneity in study designs and outcomes, a narrative synthesis was conducted. Results: Nineteen studies met the inclusion criteria. Palliative care interventions consistently reduced dyspnea, fatigue, anxiety and depression and were associated with improved functional status and QoL. Integrated palliative–cardiology programs were linked to fewer hospital readmissions, shorter hospital stays and increased documentation of advance care planning. However, methodological variability, small sample sizes and non-standardized outcome measures limited comparability across studies. Conclusions: The evidence supports the early incorporation of palliative care into routine management of chronic heart failure. Early, multidisciplinary and patient-centered approaches enhance clinical and psychosocial outcomes while improving healthcare efficiency and ensuring that care aligns with patients’ goals, values and quality-of-life priorities.

## 1. Introduction

Chronic heart failure (CHF) is a global health problem affecting more than 64 million people worldwide [1,2]. It is characterized by a progressive decline in cardiac function, frequent exacerbations, and a high risk of mortality [3,4]. Despite advances in the medical field and device-based therapies, patients with CHF continue to experience disabling symptoms such as dyspnea, fatigue, anxiety, and depression, which impair their QoL and lead to recurrent hospitalizations [5,6,7,8]. Recent research has highlighted the growing importance of prioritizing QoL rather than aggressive life-prolonging treatments in advanced heart failure [9]. Subsequent studies further explored the ethical and clinical implications of these approaches [10], emphasizing the role of palliative care in supporting goal-concordant decisions during late stages of disease [11].

Standard heart failure management focuses primarily on optimizing hemodynamics, preventing disease progression, and reducing mortality [3,4]. Although evidence-based therapies have improved survival chances, they do not fully address the multidimensional needs of patients [12,13,14]. Symptom relief, psychological support, and assistance with complex decision-making often remain unmet [15,16,17]. As the disease advances, patients and families are faced with difficult choices regarding device implantation, hospitalizations, and end-of-life care, which are not always supported by structured discussions or holistic interventions, leading to a fragmented care delivery and poor symptom management [18,19,20].

Palliative care aims to improve QoL through the relief of symptoms, psychosocial support, and assistance with advance care planning [21,22]. Traditionally associated with oncology and end-of-life care, palliative care has gradually expanded to non-malignant chronic conditions, including heart failure [23,24,25]. Early integration of palliative care into the trajectory of heart failure has been proposed as a strategy to reduce symptom burden, improve both patient and caregiver satisfaction, and optimize the use of all healthcare resources available [5,6,8,26].

Although several randomized trials, observational studies, and qualitative reports have investigated palliative care interventions in heart failure, the evidence remains fragmented [27,28]. Previous reviews have highlighted potential benefits, but results are inconsistent due to heterogeneity in populations, interventions, and outcome measures [29,30]. Moreover, palliative care remains underutilized in heart failure compared to oncology and exhibits considerable heterogeneity across different countries and regions, reflecting a lack of standardized frameworks and uniformity in practice globally [31,32]. A clear synthesis of the available evidence is therefore essential [27,29,30].

The aim of this systematic review is to evaluate the impact of palliative care interventions in chronic heart failure on four key domains: symptom burden, QoL, healthcare utilization, and decision-making at the end of life. By providing an updated and structured synthesis of available studies, this review seeks to clarify the role of palliative care in the management of chronic heart failure and highlight areas for future research and clinical practice improvement [6,8,26,33].

## 2. Materials and Methods

### 2.1. Protocol and Registration

This systematic review was conducted in accordance with the Preferred Reporting Items for Systematic Reviews and Meta-Analyses (PRISMA 2020) statement [34,35,36]. The review protocol was prospectively developed to ensure methodological transparency, reproducibility, and adherence to international reporting standards [37].

All methodological steps—including the definition of eligibility criteria, formulation of the research question using the Population, Intervention, Comparator, Outcome (PICO) framework, development of the search strategy, data extraction templates, and risk-of-bias assessment tools—were pre-specified prior to the initiation of the literature search [38,39,40].

This review summarizes current evidence on the overall impact of palliative care interventions in chronic heart failure [5,6,7,8,26,33].

The protocol was prepared following PRISMA-P recommendations and has been registered in the International Prospective Register of Systematic Reviews (PROSPERO, ID 1172739) [37,41]. All procedures were conducted in accordance with PRISMA 2020 methodological standards to ensure transparency and reproducibility [34,35,36].

The completed PRISMA 2020 checklist is available in the Supplementary Materials and online at https://www.prisma-statement.org/ (accessed on 10 October 2025).

### 2.2. Eligibility Criteria

Studies were included based on predefined eligibility criteria developed according to the PICO framework to ensure methodological consistency [42].

The population consisted of adult patients (≥18 years) diagnosed with chronic heart failure, regardless of ejection fraction subtype (HFrEF or HFpEF) [15,21,43], functional class (NYHA II–IV) [21,22], or care setting (inpatient, outpatient, or community-based) [5,6,8,18].

The intervention of interest was any structured palliative care approach, including inpatient or outpatient consultations, multidisciplinary programs, early integration models, hospice care, or advance care planning initiatives [29,30,44].

The comparator was usual or standard heart failure management, or alternative non-palliative interventions when applicable [21,22,24,27].

Eligible outcomes included studies reporting at least one of the following:

1. Symptom burden (e.g., dyspnea, fatigue, anxiety, depression) [26,31];
2. QoL (measured using validated instruments such as Kansas City Cardiomyopathy Questionnaire (KCCQ), Minnesota Living with Heart Failure Questionnaire (MLHFQ), EuroQol Five-Dimension Questionnaire (EQ-5D), or Short Form Health Survey 36 (SF-36)) [33,45];
3. Healthcare utilization (hospitalizations, emergency department visits, length of stay) [36,37];
4. Decision-making or end-of-life outcomes (advance care planning, Do Not Resuscitate (DNR) documentation, Implantable Cardioverter-Defibrillator (ICD) deactivation, hospice referral) [29,30,44];
5. Mortality (if reported as a secondary outcome) [18,19,36].

Eligible study designs included randomized controlled trials (RCTs), prospective or retrospective cohort studies, cross-sectional studies, and systematic reviews or meta-analyses providing original data [33,39]. Qualitative studies were included when they provided relevant insights into patient or caregiver experiences [28,46].

Studies were excluded if they involved pediatric populations; consisted of case reports, editorials, or expert opinions without primary data [33,47]; focused exclusively on oncologic or surgical cohorts without a heart failure subgroup [4,16,24]; or were non-English publications or published before 1 January 2005 [1,3,39].

### 2.3. Information Sources and Search Strategy

A comprehensive and systematic literature search was performed to identify relevant studies evaluating the impact of palliative care in patients with chronic heart failure [1,2,3,4,16,33].

The following electronic databases were searched: PubMed/MEDLINE, Scopus, and Web of Science (Core Collection) [48,49,50]. The search covered the period from 1 January 2005 to 1 March 2025, and was limited to studies published in English [1,3,39].

Additional sources, including the reference lists of retrieved articles and relevant review papers, were screened manually to ensure completeness [33,39].

The search strategy combined controlled vocabulary (Medical Subject Headings, MeSH) and free-text terms related to heart failure and palliative care [50]. The main PubMed search string was as follows: (“Heart Failure” [Mesh] OR “heart failure” [tiab] OR “cardiac failure” [tiab] OR HFrEF [tiab] OR HFpEF [tiab]) AND (“Palliative Care” [Mesh] OR palliative [tiab] OR “supportive care” [tiab] OR hospice [tiab] OR “end-of-life” [tiab] OR “advance care planning” [tiab] OR ACP [tiab]) AND (english [lang]) AND (“1 January 2005” [Date-Publication]:“1 March 2025” [Date-Publication])

Equivalent search terms were adapted for Scopus and Web of Science according to their specific syntax [48,49,50].

The search results were exported into EndNote Version 21 reference management software for organization and duplicate removal before screening [51].

### 2.4. Study Selection and Data Extraction

All retrieved citations were imported into EndNote reference management software, and duplicate records were removed prior to screening [51,52,53].

The study selection process was conducted in two sequential stages [20,21,33,53]. In the first stage, titles and abstracts were screened independently by two reviewers to assess eligibility based on the predefined inclusion and exclusion criteria [33,39,50,53]. Articles deemed potentially relevant were retrieved for full-text evaluation. Disagreements between reviewers were resolved through discussion and consensus, or by consultation with a third reviewer when necessary [53,54]. In the second stage, full-text articles were reviewed in detail to confirm eligibility [1,3,39].

The selection process and the number of studies excluded at each step were documented using the PRISMA 2020 flow diagram [20,21,33,55]. Reasons for exclusion at the full-text stage were recorded and categorized (e.g., population, intervention, outcome, study design) [39,50].

For each study included, data were extracted independently by two reviewers using a standardized data extraction form developed according to PRISMA-P recommendations [55,56]. Extracted variables included bibliographic information (author, year, country, journal); study design and setting; sample size and patient characteristics; type and timing of palliative care intervention; comparator (if applicable); outcome measures and main findings (symptom burden, QoL, healthcare utilization, decision-making, mortality); statistical methods and key results; and reported limitations and potential conflicts of interest [50,56]. Any discrepancies in data extraction were discussed and resolved by consensus. The final dataset was reviewed for completeness and accuracy prior to synthesis [56,57]. Qualitative and quantitative findings were integrated using a narrative weaving approach, in which qualitative themes helped contextualize and interpret quantitative outcomes.

### 2.5. GRADE Approach

Certainty of evidence was assessed using the GRADE (Grading of Recommendations, Assessment, Development and Evaluation) approach, evaluating risk of bias, inconsistency, indirectness, imprecision, and publication bias across all predefined outcomes.

## 3. Results

The initial database search identified a total of 1236 records: 528 from PubMed, 412 from Scopus, and 296 from Web of Science [39,50]. After removing 214 duplicates, 1022 unique records remained for title and abstract screening [58].

Following the first screening phase, 67 full-text articles were assessed for eligibility. Of these, 48 studies were excluded for reasons such as:

- non-heart failure population (*n* = 14);
- lack of palliative care intervention (*n* = 11);
- non-original data (*n* = 10);
- publication before 2005 (*n* = 7); and
- incomplete outcome reporting (*n* = 6) [16,39,59].

**Figure 1:**
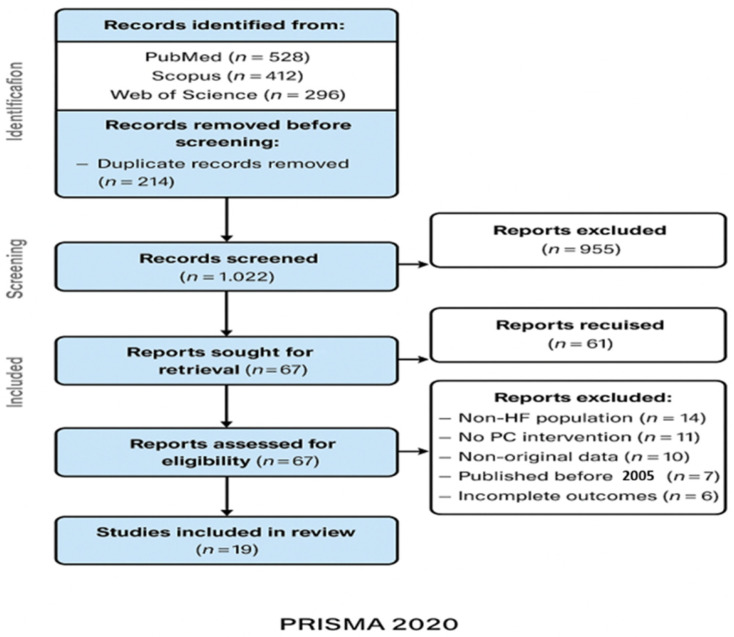
PRISMA 2020 flow diagram illustrating the process of study identification, screening, eligibility assessment, and final inclusion in the systematic review.

Finally, 19 studies met all inclusion criteria and were included in the systematic review [1,2,3,4,5,6,7,8,9,10,11,12,13,14,15,16,17,18,19,35,36,60]. The study selection process is illustrated in the PRISMA 2020 flow diagram (Figure 1) [20,21,33,61].

### 3.1. Characteristics of Included Studies

The 19 included studies comprised: 2 RCTs [2,5,8]; 6 cohort studies (prospective or retrospective) [18,19]; 3 systematic or integrative reviews [1,3,4]; 2 large database analyses [12,18,62]; and 6 qualitative or mixed-method studies [28,43,61]. Sample sizes ranged from 25 to 40,000 participants, reflecting a wide variation in study scope and methodology [18,19,63]. Most studies were conducted in North America (*n* = 10) and Europe (*n* = 6), with additional contributions from Asia (*n* = 3) [14,16,31].

Interventions included structured inpatient or outpatient palliative care consultations, multidisciplinary integrated programs, hospice care, and advance care planning (ACP) initiatives [45,57,64,65].

The duration of follow-up varied between 3 months and 2 years, depending on study design and setting [17,66]. A detailed summary of the characteristics of the included studies, including population, intervention type, outcomes assessed, and main findings, is presented in Table 1 [66,67].

For clarity, the 19 included studies were narratively grouped according to intervention type and care setting. Integrated or multidisciplinary programs, including nurse-led, family-focused, or cardiology–palliative collaborations, were evaluated in studies [65,71]. Early or structured outpatient palliative consultations and referral models were analyzed in [64,65,69], while in-hospital or hospice-based interventions were described in [69,70,72]. In addition, systematic reviews and meta-analyses providing aggregated evidence across diverse models were included as overarching sources [28,68]. This categorization enhances clarity and allows for context-specific comparisons of outcomes related to symptom burden, QoL, healthcare utilization, and end-of-life decision-making.

### 3.2. Risk of Bias Assessment

The risk of bias for the included studies was assessed independently by two reviewers using validated and study-design-specific tools [58,59,60]. For RCTs, the Cochrane Risk of Bias 2.0 (RoB 2.0) tool was applied, evaluating five key domains: randomization process, deviations from intended interventions, missing outcome data, measurement of outcomes, and selective reporting [58,59].

For observational studies, the ROBINS-I (Risk of Bias in Non-randomized Studies of Interventions) instrument was used, assessing confounding, selection bias, classification of interventions, deviations from intended interventions, missing data, and outcome measurement [35,59,60]. Qualitative studies were appraised using the Joanna Briggs Institute (JBI) Critical Appraisal Checklist, which considers methodological congruence, data representation, and researcher reflexivity [61].

Each domain was rated as having low, moderate, or high risk of bias, and an overall judgment was determined for each study [59,60,61]. Discrepancies between reviewers were resolved through discussion and consensus, or by consulting a third reviewer when necessary [63].

To ensure transparency, the results of the bias assessment were summarized narratively and graphically. The overall distribution of bias judgments across domains is presented in Table 2 [58,59,60,61,63]. The overall methodological quality of the included evidence was interpreted in the context of study design, sample size, and reporting clarity [1,3,4,16,33,63].

### 3.3. Symptom Burden

Across the included studies, palliative care interventions were consistently associated with a reduction in symptom burden among patients with chronic heart failure [1,2,3,4,5,8,9,17,26,31,36,69]. Most randomized controlled trials and observational studies reported significant improvements in the control of dyspnea, fatigue, anxiety, and depression following palliative care involvement [2,5,8,9,17,31,69].

In the randomized controlled trial by Bekelman et al. (2018), patients who received early palliative care consultations demonstrated significantly lower symptom scores on the Edmonton Symptom Assessment Scale (ESAS) and improved overall well-being compared with standard care [5]. Similarly, the trial by Sidebottom et al. (2015) found a marked reduction in depressive symptoms and anxiety, accompanied by a clinically meaningful increase in quality-of-life scores [26].

Qualitative findings from Hupcey et al. (2009) further supported these outcomes, with patients describing relief from persistent breathlessness, better emotional coping, and a greater sense of control over their illness after engagement with palliative care services [9]. In the longitudinal study by Fried et al. (2007), symptom trajectories indicated progressive improvement in dyspnea and fatigue during follow-up among patients receiving integrated palliative programs [17].

The systematic reviews by Diop (2017) and Kim (2022) corroborated these findings, emphasizing that palliative care reduced the severity and frequency of both physical and psychological symptoms across diverse healthcare settings [73]. The observed benefits were consistent across inpatient, outpatient, and community-based interventions, suggesting that symptom relief represents one of the most robust and reproducible effects of palliative care in chronic heart failure [2,3,4,5,8,9,17,31,74].

The detailed GRADE approach for symptom burden is presented in Supplementary Table S1.

### 3.4. Quality of Life (QoL)

Improvement in QoL emerged as one of the most consistent outcomes across the included studies [2,3,4,5,8,9,17,31,32,33,74]. Twelve of the nineteen studies reported statistically or clinically significant enhancements in QoL following palliative care interventions [2,5,8,17,27,32,33,74]. The positive effects were observed across a variety of settings, including outpatient programs, integrated hospital-based consultations, and community follow-up models [2,5,8,9,15,27,74].

In the randomized controlled trial conducted by Bekelman et al. (2018), patients who received early palliative care achieved a mean increase of 15 points in the KCCQ score compared with standard care, reflecting a substantial improvement in functional status and overall well-being [5]. Similarly, Sidebottom et al. (2015) demonstrated significant gains on the MLHFQ and decreased depression scores over six months of follow-up [26].

The long-term follow-up trial by Brännström et al. (2014) confirmed that the benefits of integrated cardiology–palliative care programs persisted up to 12 months, maintaining stable QoL levels even as disease severity progressed [6]. Systematic reviews by Kim et al. (2022) and Kavalieratos et al. (2016) further consolidated these results, highlighting moderate-to-large effect sizes in pooled analyses, especially in multidimensional QoL instruments capturing both physical and psychosocial domains [8,12].

Qualitative studies, such as those by Hupcey et al. (2009) and Timonet-Andreu et al. (2018) provided complementary insights, describing enhanced emotional resilience, greater patient–family communication, and an improved sense of dignity and autonomy among participants receiving palliative care [9,28].

Taken together, these results underscore that integrating palliative care into the management of chronic heart failure substantially enhances patients’ QoL across both clinical and psychosocial dimensions [2,3,4,5,8,9,13,17,31,32,33,70,74,75,76].

The GRADE certainty assessment for quality of life is shown in Supplementary Table S2.

### 3.5. Healthcare Utilization

Several studies demonstrated that the integration of palliative care in chronic heart failure management was associated with a reduction in healthcare utilization, particularly regarding hospital readmissions, emergency department visits, and total length of stay [2,4,6,7,12,18,19,36,37,71]. The results were consistent across multiple study designs and healthcare systems [4,6,12,18,19,36,71].

In the multicenter observational study by Whellan et al. (2014), palliative care consultations were linked to a 25% reduction in hospital readmissions and a higher rate of documented DNR orders [62]. Similarly, the retrospective cohort study by Gelfman et al. (2018) reported a significant decline in both hospitalizations and emergency department (ED) visits following inpatient palliative care consultation [19].

Large database analyses provided additional evidence for system-level impact. Cheng et al. (2014) showed that in-hospital palliative consultations were associated with lower in-hospital mortality and shorter length of stay, although only 5% of patients with heart failure received such care [69]. In a nationwide study, Warraich et al. (2018) observed an increasing trend in palliative care utilization among heart failure patients in the United States, but overall referral rates remained below 10%, indicating persistent underuse [18].

Internationally, similar results were reported by Guo et al. (2018) in China and Aldridge et al. (2016) in Europe, where facilities with established palliative teams experienced fewer readmissions and shorter hospital stays compared to centers without integrated services [18,30]. The evidence was supported by meta-analyses, such as Kavalieratos et al. (2016), which found moderate evidence that palliative interventions reduced acute healthcare utilization without compromising survival [12]. Overall, the evidence indicates that early or structured palliative care interventions not only improve symptom control and QoL but also contribute to more efficient and patient-centered healthcare use in chronic heart failure [2,4,6,7,10,12,14,18,19,36,37,75].

Certainty of evidence for healthcare utilization is provided in Supplementary Table S3.

### 3.6. Decision-Making and End-of-Life Care

Palliative care interventions were also found to play a significant role in improving communication, ACP, and decision-making in patients with chronic heart failure [13,14,15,29,30,45,57,77]. Across the included studies, enrollment in palliative care programs was consistently associated with earlier initiation and higher quality of advance care planning and end-of-life discussions, increased documentation of care preferences, and greater involvement of patients and families in treatment decisions [2,5,6,9,45,77].

In the multicenter study by Whellan et al. (2014), the implementation of a dedicated palliative care service led to a substantial increase in DNR documentation and advance directive completion compared with standard cardiology care [62]. Similarly, Bekelman et al. (2018) reported that patients receiving early palliative consultations were more likely to participate in ACP conversations and have documented care goals aligned with their values and prognosis [5].

Qualitative investigations further illustrated the emotional and relational impact of these interventions. In Timonet-Andreu et al. (2018), family-centered palliative care models fostered more open communication and greater satisfaction with care among caregivers, who felt better supported in end-of-life decision-making [28]. Hupcey et al. (2009) and Fried et al. (2007) likewise described that palliative involvement reduced decisional conflict and improved patients’ sense of control over their care trajectories [9,17].

At the health system level, Crespo-Leiro et al. (2018) and Aldridge et al. (2016) found that institutions with integrated palliative services had higher rates of documented ACP and hospice referrals, alongside fewer aggressive interventions during terminal hospitalizations [30,32].

Overall, these findings indicate that palliative care facilitates goal-concordant care and supports ethical, patient-centered decision-making throughout the continuum of heart failure management [29,30,45,57,76,77,78].

The GRADE assessment for decision-making outcomes is available in Supplementary Table S4.

### 3.7. Summary of GRADE Assessment

A concise GRADE assessment indicated moderate certainty for symptom burden, quality of life, and decision-making outcomes, and low certainty for healthcare utilization and mortality (Table 3).

The certainty of evidence for all predefined outcomes was evaluated using the Grading of Recommendations, Assessment, Development and Evaluation (GRADE) approach. Symptom burden and quality of life were rated as having moderate certainty, reflecting consistent improvements across randomized and observational studies with only limited downgrading for inconsistency and methodological concerns. Evidence for healthcare utilization was judged as low certainty due to heterogeneity across study designs, reliance on non-randomized data, and imprecision in effect estimates. Certainty regarding decision-making and end-of-life communication outcomes was considered moderate, although variation in care models and measurement tools required minor downgrading. Mortality demonstrated low to very low certainty because findings were inconsistent and confidence intervals frequently crossed the null. Overall, the GRADE assessment supports the robustness of benefits observed in symptoms, quality of life, and decision-making, while highlighting the need for higher-quality randomized evidence in utilization and survival outcomes.

## 4. Discussion

### 4.1. Overview of Main Findings

This systematic review provides an updated synthesis of the evidence on the impact of palliative care in chronic heart failure. Across a diverse body of randomized trials, observational cohorts, qualitative studies, and reviews, palliative care interventions consistently showed beneficial effects on clinically relevant and patient-centered outcomes [13]. Rather than reiterating the detailed results, the key finding is that these benefits were observed across heterogeneous models of care and different healthcare settings, suggesting that the positive impact of palliative care is robust and not limited to a single delivery approach [17].

Symptom relief remains one of the most consistent outcomes, particularly for dyspnea, fatigue, anxiety, and depression—symptoms that frequently persist despite optimized cardiology management [27]. The convergence of evidence across early integrated consultations, multidisciplinary collaborations, and hospice-based models indicates that these improvements are likely mediated by timely symptom assessment, better communication, and proactive advance care planning. These mechanisms are clinically important because they address dimensions of suffering not adequately targeted by conventional heart failure therapies [29].

Quality of life improvements were similarly consistent across studies using validated instruments such as the KCCQ and MLHFQ [31]. The durability of these effects over time suggests that palliative care supports long-term psychosocial stability through interdisciplinary interventions and repeated discussions about values and goals [32]. This points to a broader therapeutic role beyond symptom control, reinforcing the relevance of palliative care for functional and emotional well-being [33].

At the health system level, the observed reductions in hospital readmissions, emergency visits, and length of stay highlight the potential for palliative care to reduce avoidable acute care use [77]. These findings have important policy implications: structured communication, early identification of symptom exacerbations, and coordinated interventions appear to drive both clinical improvements and greater efficiency in care delivery [73,78].

Palliative care also plays a pivotal role in decision-making, facilitating earlier and more meaningful end-of-life discussions and ensuring that treatment aligns with patient preferences [45]. These effects underscore its importance in supporting ethical, informed, and patient-centered decisions throughout the heart failure trajectory [57].

Taken together, the evidence indicates that palliative care should be considered a core component of comprehensive heart failure management [77]. Conventional cardiology remains primarily focused on hemodynamic optimization, device therapy, and survival; however, these strategies do not fully address the multidimensional needs of patients. By contrast, palliative care provides longitudinal, holistic support that complements disease-directed treatments and enhances both clinical outcomes and quality of life [73].

### 4.2. Comparison with Previous Literature

The findings of this review are consistent with and extend previous evidence demonstrating the beneficial role of palliative care in chronic heart failure. Earlier meta-analyses, such as that by Kavalieratos et al. (2016), showed improvements in symptom control and quality of life but were limited by small sample sizes and heterogeneous interventions [12]. The present synthesis incorporates more recent studies conducted across different care settings and geographical regions, strengthening the overall certainty of benefit and suggesting that the effectiveness of palliative care is reproducible across diverse healthcare contexts [14,15].

The results also align with contemporary heart failure guidelines from the European Society of Cardiology and the American Heart Association, both of which emphasize early integration of palliative care as part of comprehensive management [57]. These guidelines highlight the importance of combining optimized medical therapy with psychosocial support, communication, and shared decision-making—elements consistently reinforced by the studies included in this review [79].

Importantly, our synthesis expands on previous literature by demonstrating that palliative care not only improves patient-reported outcomes but also has meaningful effects at the health-system level [78]. Reductions in readmissions and acute care use observed in this review mirror findings from recent large-scale analyses, underscoring the potential for palliative care to improve efficiency and reduce avoidable utilization within cardiovascular services. These system-level effects support the emerging view that palliative care is not an adjunct but an essential component of value-based heart failure care [73].

Qualitative evidence further complements these conclusions by illustrating the relational and emotional dimensions of palliative care. Studies consistently report better communication, greater family involvement, and enhanced decision-making confidence among patients receiving palliative support [45]. These observations help explain why palliative interventions improve both psychological well-being and satisfaction with care, reinforcing the importance of integrating relational and communication-focused elements into routine cardiology practice [77].

Overall, this synthesis supports a growing international consensus that palliative care offers multidimensional benefits—clinical, emotional, and organizational [73]. By contextualizing these findings within guideline recommendations and recent health-system data, the current review provides a clearer understanding of how palliative care contributes to modern, evidence-based heart failure management and highlights the need for broader implementation efforts across different healthcare environments [79,80].

### 4.3. Clinical Implications

The findings of this systematic review have several important implications for clinical practice and healthcare policy [21,22,36,57,73,79,80].

First, they emphasize that palliative care should not be reserved solely for end-stage disease, but rather be integrated early in the trajectory of chronic heart failure [5,6,8,15]. Early referral enables proactive symptom control, improved communication, and shared decision-making—elements that contribute to better long-term outcomes and reduced emotional distress for both patients and caregivers [29,30,45,77].

Second, the evidence underscores the need for multidisciplinary collaboration between cardiology, internal medicine, and palliative care specialists [21,22,57,73,79]. Integrated models of care have been shown to produce the most significant improvements in QoL and healthcare efficiency [18,19,36,37,73]. Such models facilitate individualized treatment goals, align therapeutic interventions with patients’ values, and prevent unnecessary hospitalizations [18,19,36,37].

Third, the consistent improvement in ACP and documentation of preferences indicates that palliative care can guide clinicians toward more ethical, patient-centered decision-making [29,30,45,57,77]. Structured ACP discussions help avoid overly aggressive interventions at the end of life and ensure that medical care reflects patients’ wishes and autonomy [6,9,13,17].

From a health system perspective, the reduction in hospitalizations and acute care use associated with palliative care implies substantial economic and organizational benefits [18,19,36,37,73]. Health systems implementing early palliative interventions for heart failure could achieve both better patient outcomes and cost savings by decreasing unplanned admissions and improving continuity and coordination of care [4,6,12].

Finally, in regions such as Eastern Europe, where palliative services remain underdeveloped, the evidence highlights the urgent need to expand training and institutional support for palliative care integration within cardiology departments [44,57,63,81]. Beyond Eastern Europe, low- and middle-income countries face substantial structural barriers to palliative care implementation, including scarce specialized services, inadequate reimbursement mechanisms, weak policy support, and limited infrastructure. These constraints markedly reduce opportunities for early referral and integrated cardiology–palliative models, despite clear evidence of benefit. Addressing these disparities is essential for achieving equitable global integration of palliative care in chronic heart failure [12,44,57]. Despite strong evidence of benefit, palliative care remains underutilized in heart failure due to clinician misconceptions regarding its role, late-stage referral patterns, and insufficient institutional or policy support. Implementation frameworks emphasizing early integration, structured referral pathways, interdisciplinary teamwork, and targeted clinician training appear most effective for improving adoption and aligning practice with guideline recommendations. Strengthening interdisciplinary education, establishing hospital-based palliative care teams, and incorporating palliative competencies into postgraduate curricula could substantially improve QoL for thousands of patients with chronic heart failure [63,73,81,82].

### 4.4. Limitations

This review has several limitations that should be acknowledged when interpreting the findings.

First, there was considerable heterogeneity among the included studies in terms of design, intervention type, and outcome measurement. This heterogeneity limited the certainty of conclusions across outcome domains, particularly for mortality and healthcare utilization, where findings were inconsistent and often based on small or heterogeneous samples [12,16,74]. The palliative care interventions ranged from single inpatient consultations to complex multidisciplinary programs, making direct comparisons challenging [5,6,67]. Variability in follow-up duration and in the instruments used to assess QoL (e.g., KCCQ, MLHFQ, EQ-5D) may have contributed to inconsistent reporting of effect sizes across studies [8,16].

Second, the number of RCTs remains limited, and many studies were observational or qualitative in nature. Although these designs provide valuable real-world insights, they are more susceptible to confounding and selection bias [9,12,13]. Consequently, the overall certainty of evidence for some outcomes—particularly healthcare utilization and mortality—should be interpreted with caution [12,16].

Third, most of the included research originated from high-income countries, particularly the United States and Western Europe. Evidence from low- and middle-income regions, where access to palliative care is more restricted, remains scarce [31,81,83]. Therefore, the generalizability of the current findings to diverse healthcare systems and cultural contexts may be limited [81,83].

Fourth, publication bias cannot be excluded. Studies demonstrating positive outcomes are more likely to be published, which may overestimate the apparent benefits of palliative care [12,84]. Although a formal assessment of publication bias was planned using funnel plots and Egger’s regression test, the small number of homogeneous studies per outcome limited the feasibility of this analysis.

Finally, while efforts were made to follow the PRISMA 2020 methodology and ensure comprehensive data extraction, language restrictions (English-only inclusion) and the exclusion of gray literature could have led to omission of relevant studies [34,38].

Despite these limitations, the overall consistency of findings across multiple designs and settings strengthens the validity of the conclusions and highlights the growing evidence base supporting palliative care in chronic heart failure [12,16,73].

### 4.5. Future Directions

Future research should aim to address several important gaps identified in this review.

First, there is a need for large, multicenter RCTs that evaluate standardized palliative care interventions in patients with chronic heart failure [16,74]. Most existing studies are small and heterogeneous, which limits the strength and generalizability of the evidence [12]. Standardizing intervention protocols and outcome measures—particularly those related to symptom burden, QoL, and healthcare utilization—would facilitate meaningful comparisons and future meta-analyses [74].

Second, future work should explore optimal timing and delivery models for integrating palliative care into heart failure management [15,17,36,85]. Evidence suggests that early involvement yields greater benefits than late referrals, yet the ideal threshold for referral remains undefined [12,21,22]. Studies comparing early versus delayed integration or evaluating stepwise referral models embedded in cardiology services could help identify best practices for implementation [21,22].

Third, there is an urgent need to include diverse healthcare settings and underrepresented populations, especially in low- and middle-income countries where access to palliative care remains limited. Cross-national studies may help identify cultural and systemic barriers to implementation and inform policy development for equitable care delivery [81,83,86].

Fourth, future investigations should incorporate patient-reported outcomes (PROs) and caregiver perspectives more systematically. Qualitative and mixed-methods research can provide deeper insights into patients’ lived experiences, emotional needs, and satisfaction with palliative interventions—elements often underrepresented in quantitative studies [9,13].

Finally, advancements in digital health and telemedicine offer new opportunities to extend palliative care beyond hospital settings. Evaluating the effectiveness of remote symptom monitoring, virtual consultations, and tele-palliative programs could transform accessibility and continuity of care for patients with chronic heart failure [48,49].

In summary, expanding the evidence base through high-quality, multidisciplinary research will be essential to fully integrate palliative care as a cornerstone of comprehensive heart failure management [12,21,22].

## 5. Conclusions

This systematic review demonstrates that palliative care represents a critical, yet often underutilized, component of comprehensive management in chronic heart failure. Across a wide range of study designs and healthcare settings, palliative interventions consistently improved symptom control, QoL, and patient-centered communication, while simultaneously reducing unnecessary hospitalizations and supporting ethical decision-making at the end of life.

The results highlight that palliative care should not be viewed as an adjunct to cardiology, but rather as an integral component of disease management, addressing the multidimensional needs—physical, emotional, social, and spiritual—that accompany advanced heart failure. The evidence supports the early and proactive integration of palliative approaches, which enhance both clinical outcomes and patient autonomy throughout the illness trajectory.

From a healthcare systems perspective, the incorporation of palliative care into heart failure programs can lead to greater efficiency and cost-effectiveness, aligning therapeutic intensity with patients’ goals and preferences. Importantly, the review underscores the persistent gaps in access to palliative care, particularly in low- and middle-income regions, and calls for policy-level strategies to expand specialized training and service availability.

In conclusion, palliative care in chronic heart failure provides measurable clinical and humanistic benefits, improving both the quality and the dignity of patients’ lives. Strengthening interdisciplinary collaboration between cardiology and palliative medicine should become a priority for modern cardiovascular care, ensuring that every patient with heart failure receives care that is not only effective but also compassionate and person-centered.

## Figures and Tables

**Table 1:** Characteristics of included studies.

No.	Author (Year), Country	Study Design	Sample Size	Palliative Care Intervention	Main Outcomes/Findings
1	Diop MS (2017), USA [7]	Systematic review (9 studies)	~1500	Mixed palliative care models	↓ symptoms, ↑ QoL, ↓ readmissions, no mortality effect
2	Bekelman DB (2018), USA [5]	RCT	150	Early palliative consult + follow-up	↑ QoL (KCCQ), ↓ symptoms, ↑ ACP discussions
3	Kim CM (2022), South Korea [8]	Integrative review	—	Consultations/multidisciplinary programs	↓ symptoms, ↑ QoL
4	Kavalieratos D (2016), USA [12]	Meta-analysis (16 studies)	—	Various models	↑ QoL, ↓ symptoms, mortality uncertain
5	Sidebottom AC (2015), USA [26]	RCT	232	Structured palliative program	↓ depression & anxiety, ↑ QoL
6	Whellan DJ (2014), UK [62]	Multicenter observational	300	Palliative consult	↑ DNR documentation, ↓ hospitalizations
7	Gelfman LP (2018), USA [19]	Retrospective cohort	400	In-hospital palliative consult	↓ hospitalizations & ED visits
8	Brännström M (2014), Sweden [6]	RCT follow-up	72	Integrated cardiology + palliative program	QoL benefits maintained 12 months
9	Hupcey JE (2009), USA [9]	Qualitative	40	Integrated palliative care	Patients report ↓ dyspnea, ↓ anxiety
10	Warraich HJ (2018), USA [18]	National database	40,000	Palliative care utilization analysis	Usage ↑ but <10% HF patients receive care
11	Von Schwarz ER (2020), USA [16]	Small cohort	100	Hospice care	↑ QoL, ↓ anxiety toward EoL
12	Aldridge MDP (2016), Europe [30]	Multicenter survey	—	Integrated palliative teams	↓ hospitalizations where teams exist
13	Timonet-Andreu E (2018), Spain [28]	Qualitative (families)	478	Family-focused palliative care	↑ communication & caregiver support
14	Guo P (2018), China [31]	Prospective cohort	210	Early palliative referral	↓ hospitalizations, ↑ QoL
15	Crespo-Leiro MG (2018), Europe [32]	Multicenter observational	—	Palliative services survey	Only 20% HF patients received palliative care
16	Singh T (2015), Asia [68]	Systematic review (16 studies)	—	Various palliative models	Benefits similar to Western data
17	Fried TR (2007), USA [17]	Longitudinal study	120	Integrated palliative program	↓ dyspnea, ↓ fatigue
18	Cheng RK (2014), USA [69]	Retrospective cohort	5000	In-hospital palliative consult	↓ mortality; only 5% received intervention
19	Collins S (2015), USA [70]	Retrospective cohort	600	Palliative consult	↓ ED visits post-consultation

**Table 2:** Risk of bias summary for included studies.

No.	Study ID	D1	D2	D3	D4	D5	Overall
1	Bekelman DB et al., 2018 [5]	Low	Low	Low	Some	Some	Some
2	Sidebottom AC et al., 2015 [26]	Low	Low	Low	Some	Low	Some
3	Brännström M, 2014 [6]	Low	Low	Low	Low	Low	Low
4	Gelfman LP et al., 2018 [19]	Some	Some	Low	Low	Low	Low
5	Whellan DJ et al., 2014 [62]	Some	Low	Low	Low	Some	Some
6	Cheng RK et al., 2014 [69]	Some	Some	Low	Low	Low	Some
7	Aldridge MDP et al., 2016 [30]	Some	Low	Low	Low	Low	Low
8	Warraich HJ et al., 2018 [18]	Low	Low	Low	Low	Low	Low

Abbreviations: D1 = Randomization process; D2 = Deviations from intended interventions; D3 = Missing outcome data; D4 = Measurement of outcomes; D5 = Selection of the reported result.

**Table 3:** Summary of GRADE certainty of evidence across outcomes.

Outcome	Overall Certainty	Explanation
Symptom burden	Moderate	Consistent improvements across RCTs and cohort studies
Quality of life	Moderate	Positive effects across multiple instruments, minor inconsistency
Healthcare utilization	Low-Moderate	Mixed findings; predominantly observational evidence
Decision-making/ACP	Low-Moderate	Consistent improvements in ACP and communication

## Data Availability

No new data were created or analyzed in this study.

## Supplementary Materials

The following supporting information can be downloaded at: https://www.mdpi.com/article/10.3390/diseases13120389/s1, Table S1. GRADE assessment for symptom burden; Table S2. GRADE assessment for quality of life; Table S3. GRADE assessment for healthcare utilization; Table S4. GRADE assessment for decision-making and end-of-life care S.

## Abbreviations

The following abbreviations are used in this manuscript:

ACP	Advance Care Planning
AHA	American Heart Association
CHF	Chronic Heart Failure
DNR	Do Not Resuscitate
EQ-5D	EuroQol Five-Dimension Questionnaire
ESAS	Edmonton Symptom Assessment Scale
ESC	European Society of Cardiology
GRADE	Grading of Recommendations, Assessment, Development and Evaluation
HrpEF	Heart Failure with Preserved Ejection Fraction
HrrEF	Heart Failure with Reduced Ejection Fraction
JBI	Joanna Briggs Institute
KCCQ	Kansas City Cardiomyopathy Questionnaire
MLHFQ	Minnesota Living with Heart Failure Questionnaire
ICD	Implantable Cardioverter-Defibrillator
NYHA	New York Heart Association
PICO	Population, Intervention, Comparator, Outcome
PRISMA	Preferred Reporting Items for Systematic Reviews and Meta-Analyses
PRISMA-P	Preferred Reporting Items for Systematic Review and Meta-Analysis Protocols
PROSPERO	International Prospective Register of Systematic Reviews
RCTs	Randomized Controlled Trials
ROBINS-I	Risk of Bias in Non-randomized Studies of Interventions
RoB 2.0	Cochrane Risk of Bias 2.0 Tool
SD	Standard Deviation
SF-36	Short Form Health Survey 36
QoL	Quality of Life
