# Supplementary material for: Palliative Care in Chronic Heart Failure: A Systematic Review of Its Impact on Symptoms, Quality of Life, and Decision-Making Process
Source: Diseases. 2025 Dec 1;13(12):389. doi: 10.3390/diseases13120389 (PMC12732146; doi:10.3390/diseases13120389)
Supplement: Supplementary file 1 [file diseases-13-00389-s001.zip › GRADE.pdf]

Supplementary Materials

Supplementary Table S1. GRADE assessment for symptom burden

Outcome	No. of studies	Risk of bias	Inconsistency	Indirectness	Imprecision	Publication bias	Overall certainty	Effect summary
Symptom burden	2 RCTs + 6 cohort studies	Some concerns	Moderate	Low	Moderate	Possible	Moderate	Consistent reduction in dyspnea, fatigue, anxiety, and depression

Supplementary Table S2. GRADE assessment for quality of life

Outcome	No. of studies	Risk of bias	Inconsistency	Indirectness	Imprecision	Publication bias	Overall certainty	Effect summary
Quality of life	2 RCTs + 5 cohort studies	Some concerns	Moderate	Low	Moderate	Possible	Moderate	Improvements across KCCQ, MLHFQ, EQ-5D

Supplementary Table S3. GRADE assessment for healthcare utilization

Outcome	No. of studies	Risk of bias	Inconsistency	Indirectness	Imprecision	Publication bias	Overall certainty	Effect summary
Healthcare utilization	≥6 cohort studies	Moderate–high	Moderate	Low–Moderate	Moderate	Likely	Low–Moderate	Reduced readmissions and ED visits

Supplementary Table S4. GRADE assessment for decision-making and end-of-life care

Outcome	No. of studies	Risk of bias	Inconsistency	Indirectness	Imprecision	Publication bias	Overall certainty	Effect summary
Decision-making / EOL care	4 cohort + qualitative	Moderate	Moderate	Moderate	Moderate	Possible	Low–Moderate	Improved ACP, communication, DNR documentation
